# Exploring Initial Management Practices Related to Anterior Shoulder Dislocation in the United Kingdom and Ireland Professional Rugby Union: An Observational Online-Based Survey

**DOI:** 10.7759/cureus.82053

**Published:** 2025-04-11

**Authors:** Kieran Hawkins, Simon Kemp, Steffan Griffin, Katrine Okholm Kryger, Sam Botchey, Craig Rosenbloom

**Affiliations:** 1 Sport and Exercise Medicine, Queen Mary University of London, London, GBR; 2 Sport and Exercise Medicine, Rugby Football Union, London, GBR; 3 Sport and Exercise Medicine, Oxford University Hospitals Trust, Oxford, GBR

**Keywords:** dislocation, england, injuries, management, rugby, scotland, shoulder, treatment, wales

## Abstract

Objective

In this study, we aimed to identify current medical practice regarding shoulder dislocation pitch-side management within men’s and women’s professional rugby union in the United Kingdom (UK) and Ireland.

Methods

An online questionnaire containing 38 questions, validated by an expert panel of clinicians, was distributed to team medical personnel at 41 professional UK and Ireland rugby teams. Distribution was conducted via the Rugby Football Union and Irish Rugby Football Union to medical personnel at English and Irish professional teams.

Results

Thirty-nine completed questionnaires were received, and one response was excluded. Most responders were doctors (n=22; 58%) and physiotherapists (n=15; 39%) and worked in the Gallagher Premiership (n=14; 37%). Most responders would attempt reduction in the medical room with normal neurovascular status (n=29; 76%). Many responders would attempt medical room reduction with motor nerve and vascular impairment, respectively (n=19; 50% and n=17; 45%). Responders would have one and two attempts at reduction in 53% (n=20) and 37% (n=14) of cases, respectively, and the Spaso technique was the most common method used (n=16; 42%).

Conclusions

While the initial management practices related to anterior shoulder dislocation in professional rugby union are fairly consistent amongst pitch-side clinicians, notable variations were also observed, including reduction settings with neurovascular impairments, reduction techniques, and reduction attempt numbers.

## Introduction

The injury burden of shoulder (glenohumeral) dislocations can be significant within professional rugby union in the United Kingdom (UK). Incidence rates of shoulder dislocation/instability are 0.025 and 1.25 per 1000 player hours of training and match play, respectively [1]. Regarding dislocation/instability injury return to play, training injuries led to 157 days, and match injuries led to 81 days of player absence, respectively [1]. This injury rate has a significant impact on professional players and clubs as it is responsible for 47% of player absence from training/competition [1]. Regarding shoulder dislocation within rugby, the most common injury mechanism entails tackling [2], anterior displacement is the most common form (98%) [3], and primary dislocation refers to the first episode [4].

The management of anterior shoulder dislocations can be complex, and the injury treatment journey includes the initial pitch side management and post-reduction care, rehabilitation, as well as surgical considerations [5]. The initial pitch-side management of shoulder dislocation in professional rugby is the focus of this article, and it may be influenced by several factors, including medical guidelines, pitch-side medical courses, and research findings [4]. The primary research question posed in this study is as follows: What are the current pitch-side management practices for anterior shoulder dislocations in professional rugby union teams in the United Kingdom and Ireland?

Several UK national guidelines that are relevant to pitch-side shoulder dislocation management have been published; however, these are limited in terms of detail and may not be evidence-based [6]. National Institute for Health and Care Excellence (NICE) guidelines (2022) and British Orthopaedic Association (BOA) guidelines (2015) both recommend immediate transfer of pre-hospital dislocation patients to the emergency department (ED) and reduction in a controlled environment [7-8]. UpToDate is an evidence-based clinical guidance website, which advises consideration of early pitch-side reduction in sporting environments if capable clinicians are present [9].

Several rugby union pitch-side immediate care medical training courses have been established and mandated by national governing bodies [10-13], which are endorsed by the Faculty of Pre-Hospital Care [14]. While these provide some recommendations for shoulder dislocation pitch-side management, they may not be evidence-based [6]. The Rugby Football Union (RFU) and Irish Rugby Football Union (IRFU) offer the Pre-hospital Immediate Care in Sport (PHICIS) [10] and the Standard Approach to Field Emergencies in Rugby (SAFE) [13] courses, respectively. Regarding anterior shoulder dislocation, the PHICIS pre-course e-learning (2023) contains some general management recommendations, including player removal from play and early shoulder reduction [15], and the PHICIS course manual (2023) contains an image of the Spaso reduction technique but lacks further detail [16]. The PHICIS course lead, when contacted, stated that the topic is discussed with clinicians with prior experience, and recommendations include one medical room Spaso reduction attempt, in cases with normal neurovascular status, before hospital transfer. The SAFE course lead, when contacted, reported that shoulder dislocation management is not taught due to an assumption of experience, but brief discussions may take place between course participants (on the advanced course). This suggests that course information may vary between courses/course materials and likely contains insufficient detail regarding the complexities of pitch-side shoulder dislocation management.

Within the literature, despite considerable debate, there is a lack of clarity around numerous pitch-side initial management aspects including optimal reduction technique [17-19], reduction setting [4,19-20], reduction attempt number [4,17], optimal analgesia [21-23], radiological imaging [18-20], and post-reduction immobilisation position/duration [24-26]. In 2015, Norte et al. published guidelines regarding pitch-side sport shoulder dislocation management based on a literature review, recommending early pitch-side reduction, seven reduction methods, and one/two reduction attempts based on neurovascular status [18]. In 2017, Shah et al. offered a systematic approach to pitch-side sport shoulder dislocation management based on a literature review, recommending early pitch-side reduction, six reduction methods, and one attempt at reduction in a neurovascularly intact shoulder [4]. Yet, there still appears to be a paucity of research regarding initial pitch-side management specific to rugby union [4], a comparison of pitch-side and hospital initial shoulder dislocation management, and the effects of pitch-side shoulder dislocation initial management on player return to play [6].

The initial management of glenohumeral dislocation in athletes is particularly important as it may have a significant impact on injury outcomes [27-28]. In systematic reviews, the reduction technique used on patients has been shown to influence reduction success rates, reduction time, pain levels, and complication rates in hospital and pre-hospital settings [6,29]. The scapular manipulation method appears optimal in hospital settings (success rate: 97%, mean reduction time: 1.75 minutes, complication rate: 0%) [29]. However, further research is required to analyse additional aspects of pre-hospital initial management on injury outcomes (reduction setting and reduction attempt numbers) and the influence of initial pitch-side management practices on recurrence risk, surgical requirement, and return to play times of athletes [6].

There exist several national management guidelines that advise different pre-hospital management and reduction locations (NICE, BOA, UpToDate) [7-9], multiple pitch-side medical training courses which may contain insufficient detail regarding the complexities of pitch-side management [10-13], and numerous literature areas of debate (optimal: reduction setting/technique/attempt number, analgesia, imaging and immobilisation) [4,17-22,24-25]. Hence, there could be variation in the pitch-side management of shoulder dislocations in senior UK and Ireland professional men’s and women’s rugby union [4], which could lead to suboptimal injury outcomes and recovery of professional players [4]. In light of this, this study aimed to identify current medical practice regarding shoulder dislocation pitch-side management within UK and Ireland professional men’s and women’s rugby union. Our areas of focus include reduction site, reduction techniques, reduction attempt numbers, analgesia used, and immobilisation advised.

## Materials and methods

Study design

An online, 38-item questionnaire was developed aimed at team medical personnel (doctors, physiotherapists, physical/sports therapists) in professional UK (English, Scottish, Welsh, Northern Irish) and Ireland women’s and men’s rugby teams. Clinicians managing only the crowd, academy players, or players from a non-professional club were excluded.

The RFU defines a professional club as “a team authorised by the RFU or appropriate union, to compete in the Premiership, Championship or equivalent level of rugby union” [30]. Based on this definition, 51 professional teams were identified as the target population within the UK and Ireland (Table 1). An audit of medical provision in English rugby union clubs, via a cross-sectional survey, identified an average of 4.4 practitioners per club [31]. With a target population of 51 professional teams, the estimated target population was 224 practitioners. Survey sample size determination resulted in a target sample of 142 practitioners [32]. 

**Table 1:** Target population of medical personnel in professional UK and Ireland rugby union

Country	Governing body	Professional league	Gender of players	Number of professional teams
England	Rugby Football Union	Gallagher Premiership	Male	11
		Rugby Football Union Championship	Male	12
		Allianz Premier 15s	Female	10
Wales	Welsh Rugby Union	United Rugby Championship	Male	4
Scotland	Scottish Rugby Union	United Rugby Championship	Male	2
Northern Ireland and the Republic of Ireland	Irish Rugby Football Union	United Rugby Championship	Male	4
All	All	International competitions	Both	8
			Total	51

Ethical approval and informed consent

The ethical approval for the project was obtained in December 2021 from the Queen Mary Ethics of Research Committee. Participants confirmed that they were able to comprehend the information sheet and consented to participate in the study (the first survey question). All data was anonymised, non-identifiable and secured according to general data protection regulations (GDPR) [33].

Development and pre-testing

A literature review failed to identify any validated questionnaires regarding sport pitch-side glenohumeral dislocation management. Hence, an original questionnaire was created based on research areas of debate and dislocation pitch-side management algorithm components provided by Shah et al. [4]. Questionnaire development involved expert panel feedback. Experts were selected by identifying individuals from the authors’ professional network and represented a range of specialities, with experience relevant to professional shoulder dislocation management. The questionnaire was sent to eight experts, including consultant doctors (upper limb orthopaedic, emergency medicine, and sport and exercise medicine) and physiotherapists with experience working in professional rugby. Feedback was used to establish usability and relevance [34], as well as the face and content validity of items [35,36]. A staggered single round of feedback was obtained from experts due to practicality and feasibility, and questionnaire amendments were made based on the comments. Alterations were not fed back to the expert panel.

Respondent demographic and professional backgrounds were explored, as well as dislocation incidence information and primary and recurrent anterior dislocation management practices (Table 2). The questionnaire underwent internal validation within the authorship group to review the survey usability and technical functionality before distribution.

**Table 2:** Questionnaire content summary

Question number	Question content
1	Study information understanding confirmation and participant consent
2-11	Responder demographic and professional background information
12-13	Use of shoulder dislocation protocol/guidelines
14-18	Shoulder dislocation experience and incidence
19-29	Primary anterior dislocation management practices; reduction setting (neurovascularly intact, motor nerve impairment, vascular impairment); reduction methods; reduction attempt number; analgesia; post-reduction radiograph; post-reduction immobilisation; post-reduction follow-up
30-37	Recurrent anterior dislocation management practices; reduction setting (neurovascularly intact, motor nerve impairment, vascular impairment); reduction methods; reduction attempt number
38	Any further questions/comments

Recruitment process and description of the sample with access to the questionnaire

An open survey was used for medical personnel with the survey hyperlink/web page address. Contact was made via email with the RFU, WRU, SRU, and IRFU medical leads, explaining the project and requesting distribution to medical personnel at the relevant professional clubs. The RFU distributed an email (15/3/23) and a follow-up email (6/4/23) containing the questionnaire hyperlink, cover letter, participant information letter, and research abstract to 87 medical personnel across 35 teams. This consisted of head doctors and physiotherapists at the English professional clubs and national team clinicians. The IRFU distributed an email (16/3/23) and a follow-up email (27/3/23) containing the questionnaire hyperlink and additional information to 12 medical personnel across six teams. Medical personnel contacted were encouraged to distribute the questionnaire to medical colleagues at their club. The WRU and SRU declined to distribute the questionnaire due to staff commitments and a preference for the author to contact clubs directly, respectively. In total, the questionnaire was distributed to clinicians from 41/51 teams of the target population.

Survey administration

The survey was voluntary without any incentives and hosted on Online Surveys [37]. Responses were collected between 5/3/23 and 30/4/23. Survey questions were not randomised. Adaptive questioning was used for question 4 to separate staff by profession and obtain further job information and question 23 to ask four further questions regarding recurrent dislocation management to responders who would manage this condition differently. All survey questions were mandatory; therefore, a completeness check was not part of the survey, and all multiple-choice questions contained an ‘other’ option with a box to provide further detail. Survey responders were able to review and change answers with a back button between pages. The study was not pre-registered before development.

Preventing multiple entries from the same individual

Unique identifiers/user IDs/IP checks/log file analysis/user registration were not used during the survey. Data was reviewed by the lead author for duplications/identical survey responses.

Analysis

Following collection, descriptive data was produced within Online Surveys [37] and Microsoft Excel [38] for each respective research question. Counts and percentages were used for all questions. Medians were calculated for continuous quantitative data. Only completed questionnaires were analysed, and response times were not collected. The mean (question 11) was calculated from the 36 responses provided.

## Results

Responses

From the medical personnel contacted, 39 completed questionnaires were received. One response was excluded because it did not meet inclusion/exclusion criteria (working in senior rugby). Two responses were missing from question 11 regarding first-team player numbers. This was deemed unlikely to affect the overall result interpretation [39].

Respondents’ demographics

Most of the responders were working in England (n=35; 92%) and the Gallagher Premiership (n=14; 37%). Various professions were represented among the respondents: doctors (n=22; 58%), physiotherapists (n=15; 39%), and a sports/rehabilitation therapist (n=1; 3%; Table 3). Doctors were from a variety of specialities and training levels; physiotherapists were largely based in elite sports (n=13; 87%; Table 3). Experience of managing shoulder dislocations ranged from none to high (41+). Responders had a median of 7.5 (IQR: 7) years of experience in professional rugby and were responsible for a median of 45 (IQR: 19.5) players on matchdays (Table 3). Responders were most commonly male (n=31; 81%), aged 31-40 years (n=19; 50%; Table 3).

**Table 3:** Demographics of questionnaire responders

Characteristic	Count (n)	Percentage (%)
Gender		
Male	31	82
Female	7	18
Age group, years		
21-30	8	21
31-40	19	50
41-50	7	18
51-60	4	11
Profession		
Doctor	22	58
Physiotherapist	15	40
Sports/rehabilitation therapist	1	3
Country of work		
England	35	92
Northern Ireland	2	5
Republic of Ireland	1	3
League worked in		
Gallagher Premiership	14	37
United Rugby Championship	4	11
Rugby Football Union Championship	9	24
Allianz Premier 15s	9	24
International competition	2	5
Acute shoulder dislocations managed in career		
0	1	3
1-5	13	34
6-10	7	18
11-20	3	8
21-40	8	21
41+	6	16
Doctor's speciality		
General practice	9	41
Sport and exercise medicine	4	18
Emergency medicine	7	32
Orthopaedic surgery	1	5
Anaesthetics	1	5
Doctor's training level		
Consultant	7	32
General practitioner	7	32
Registrar (ST3-8)	5	23
General practice trainee (ST1-3)	1	5
Core trainee (CT1-CT3)	1	5
Non-training middle grade	1	5
Physiotherapist's speciality		
Elite sport	13	87
Private musculoskeletal	2	13

Usage of guidelines

**Figure 1:**
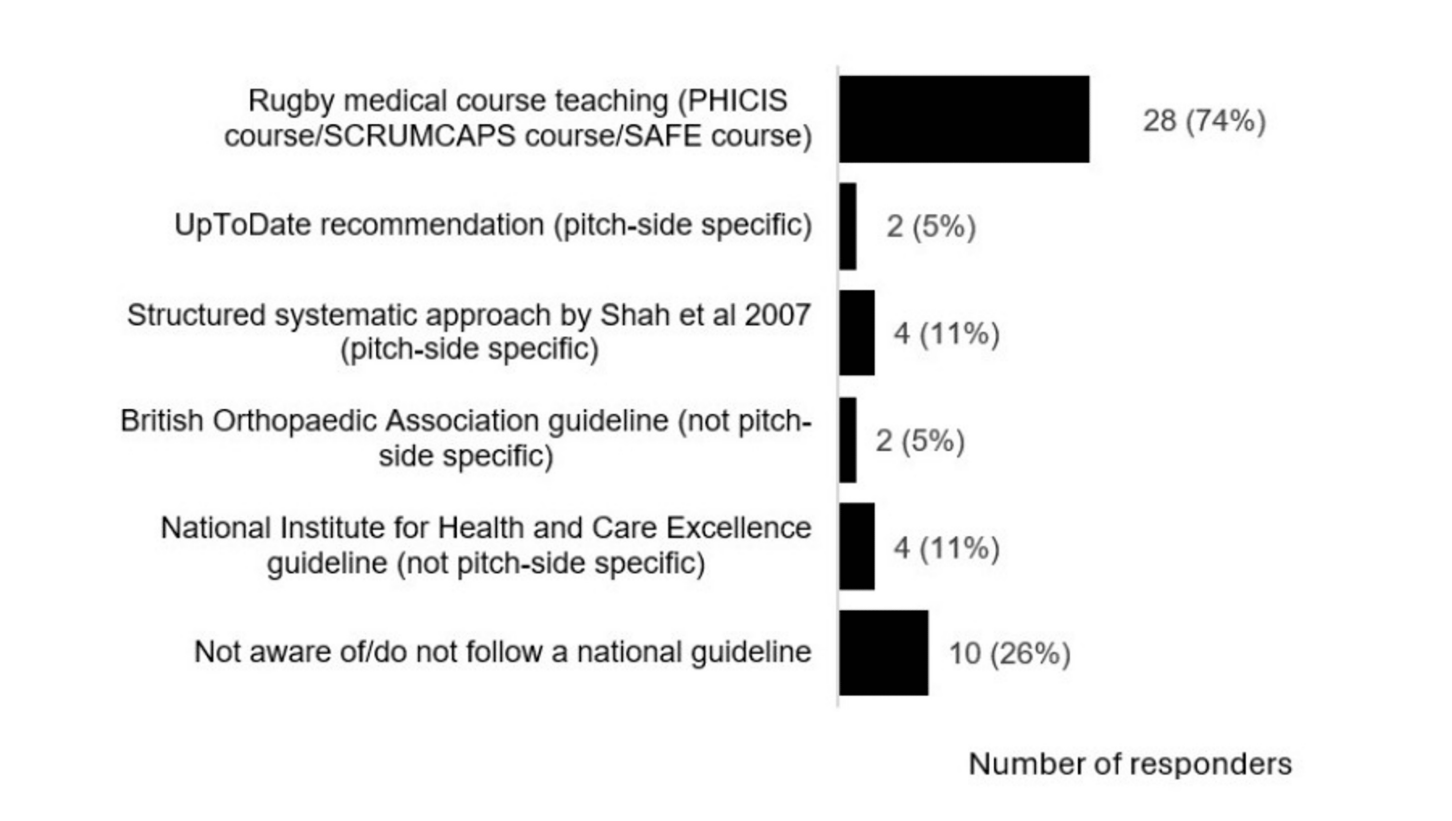
National guidelines followed by responders working in professional rugby union regarding pitch-side shoulder dislocation management* ^*^[4,7-13] PHICIS: Pre-Hospital Immediate Care in Sport; SCRUMCAPS: The Scottish Rugby Union Medical Cardiac & Pitch Side Skills; SAFE: Standard Approach to Field Emergencies in Rugby

Many responders did not have an internal club guideline for management of pitch-side shoulder dislocations (n=31; 82%). The most common national guidelines used were recommendations from practical immediate care pitch-side courses (n=28; 74%) (Figure 1). However, 24% (n=9) of responders used multiple guidelines and 26% (n=10) reported that they did not follow/were not aware of any national guidelines. 

Acute anterior primary dislocation management

**Figure 2:**
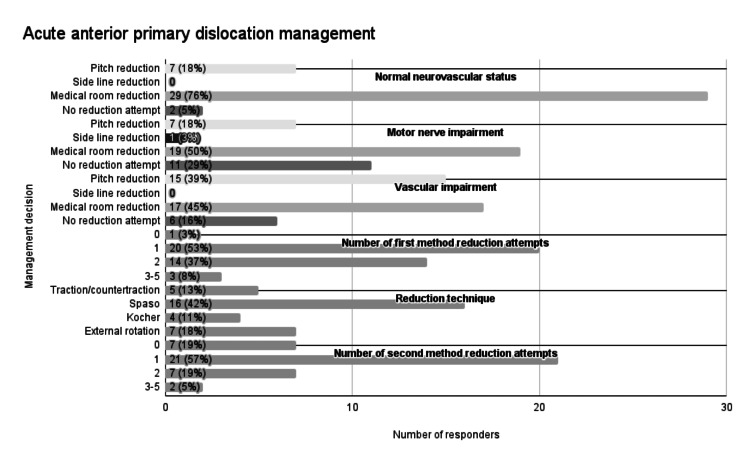
Acute anterior primary dislocation management

**Figure 3:**
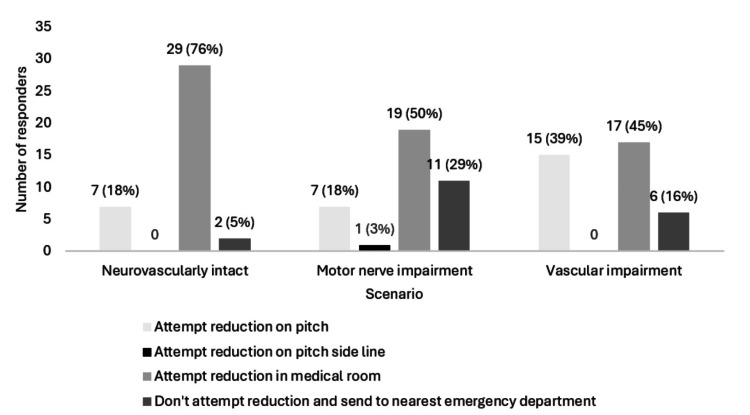
Responders' preferred primary anterior shoulder dislocation reduction location

**Figure 4:**
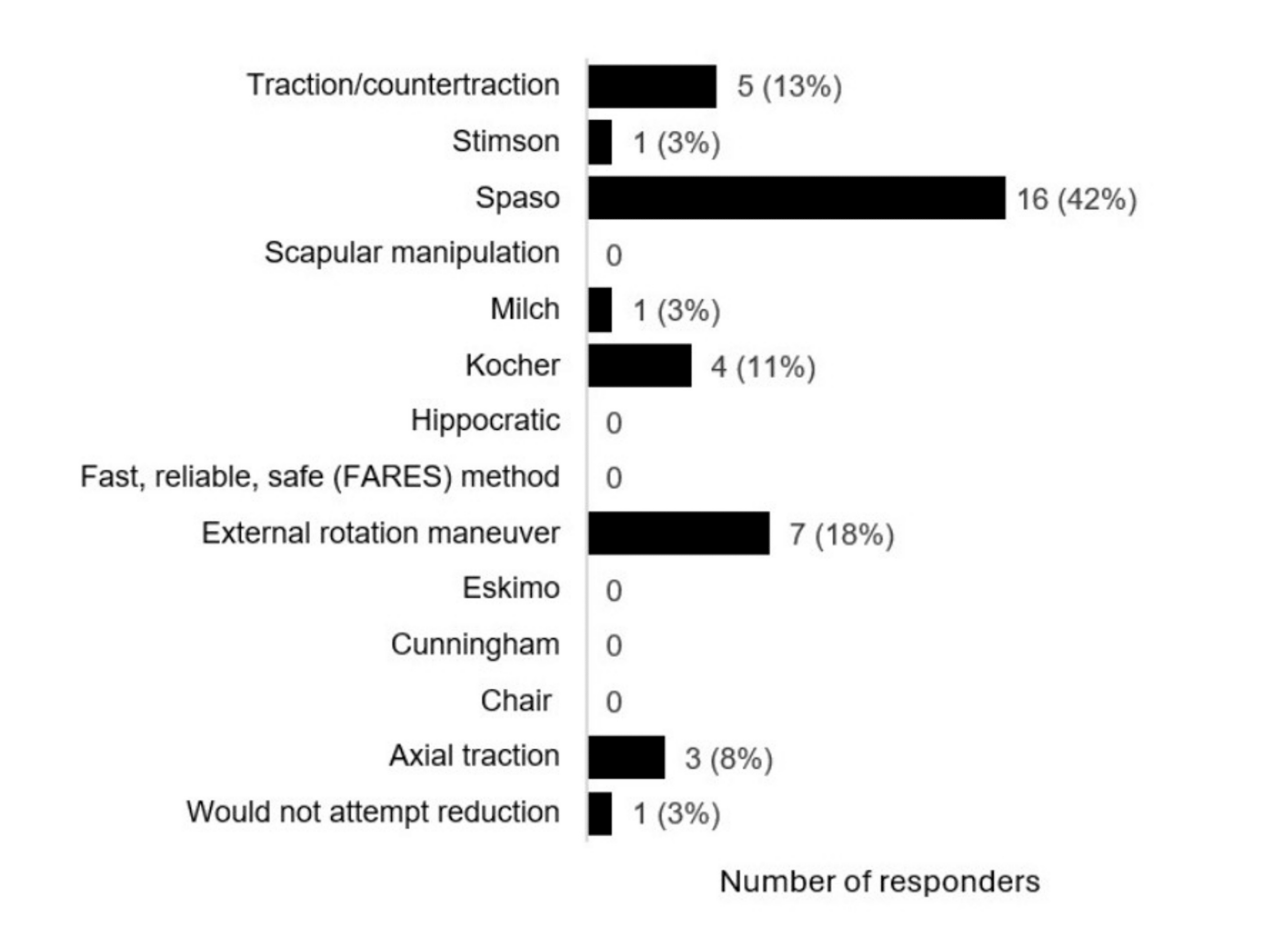
Responders' preferred initial reduction method in scenario of primary anterior shoulder dislocation* ^*^[6]

A summary of acute anterior primary dislocation management decisions is provided in Figure 2. Most responders would attempt reduction in the medical room (n=29; 76%) when a player had an anterior primary dislocation with normal neurovascular status (Figure 3). Furthermore, some responders would attempt medical room reduction when signs of motor nerve (n=19; 50%) and vascular impairment (n=17; 45%) were present (Figure 3). The number of first-method reduction attempts by medical staff varied: no attempts 3% (n=1); one attempt 53% (n=20); two attempts 37% (n=14); and three to five attempts 8% (n=3). The Spaso reduction technique was the most commonly used method (n=16; 42%; Figure 4). Some responders would not attempt a second reduction technique (n=7; 19%). Regarding responders who would attempt the second reduction method, 70% (n=21) would have one attempt, 23% (n=7) would have two attempts, and 7% (n=2) would have three to five attempts.

The most popular analgesia employed was the inhalation of Penthrox, a methoxyflurane auto inhaler [40], and Entonox, an inhaled mixture of nitrous oxide and oxygen [41], which were used by 95% (n=36) and 32% (n=12) of responders, respectively. All responders would obtain post-reduction radiograph within 24 hours: 68% (n=26) via the free public National Health Service (NHS), and 32% (n=12) via private facilities. All responders would opt to immobilise the shoulder post-reduction. Internal rotation immobilisation was the most common (n=26; 68%), followed by comfort-based immobilisation (n=11; 29%). The type of clinic follow-up advised for athletes varied: club doctor 50% (n=19); orthopaedic private 76% (n=29); orthopaedic NHS 18% (n=7); and sports medicine 3% (n=1).

Anterior recurrent dislocation management

Many responders would not change their clinical management if a player suffered an anterior recurrent shoulder dislocation (n=31; 82%). As for responders who would change their management, Spaso was the most preferred initial reduction method (n=3; 43%), with a mean of 2.6 (SD: 1.2) attempts. Traction/countertraction was the most preferred second reduction method, with a mean of 2.0 (SD: 1.6) attempts.

## Discussion

This study was designed as an exploratory investigation into pitch-side management practices for anterior shoulder dislocation within professional rugby union-a topic for which there is currently limited published data, guidance, or standardised practice. As such, a descriptive approach was intentionally adopted to provide an initial overview of current clinical behaviours across a range of professional settings. While a more detailed comparative analysis could offer further insights, we sought to avoid data-driven hypothesis testing and confirmation bias by not selectively analysing for statistical significance where the study was not powered to detect such associations. The intention was to identify key areas of variation and consistency to inform the development of future research questions. These findings may serve as a foundation for more robust, hypothesis-driven studies that could ultimately contribute to improved governance and standardisation of athlete care within rugby and potentially other collision sports.

The study objective was to identify current medical practice regarding shoulder dislocation pitch-side management within UK and Ireland professional men’s and women’s rugby union. The study identified that management practices are fairly consistent amongst pitch-side clinicians, but some variation in management practices was identified, particularly regarding reduction site (44-76% agreement), reduction technique (42% agreement), and reduction attempt number (ranged one to five). As the first study of its kind, these findings may be particularly useful for reflection and increased clarity of clinicians and pitch-side course teachers in professional rugby union.

Regarding shoulder dislocation internal club guidelines, most responders (n=31; 82%) did not have a guideline, which highlights a possible improvement area for medical teams. Regarding national guidelines, most responders (n=28; 74%) based their practice on pitch-side medical courses. This emphasises the importance of pitch-side course guidance and the vital need for courses to be evidence-based and regularly updated, including pre-course e-learning, course manual, and course discussions. A notable number of responders reported using multiple guidelines (n=9; 24%), and not being aware of/following guidelines (n=10; 26%), without qualitative data this is somewhat difficult to interpret, but could suggest clinicians have a lack of clarity regarding optimal evidence-based guidelines and pitch-side shoulder dislocation management practices.

Regarding optimal reduction site, most clinicians (n=29; 76%) opted for neurovascularly intact dislocation reduction in the medical room, showing a clear preference for on-site but non-pitch-side reduction. This is consistent with previous advice from Norte et al. [18], Shah et al. [4], and PHICIS course recommendations but contrasts with NICE (2022) and BOA (2015) guidelines, which encourage immediate transfer to an emergency department [7-8]. This discrepancy highlights an area where guideline clarification is needed. The medical room may be favoured due to its controlled, non-crowd facing environment, allowing optimal assessment, equipment, and treatment [42].

Neurovascular impairment was an area of inconsistency amongst responders. Regarding motor nerve impairment, 50% (n=19) of responders continued to opt for medical room reduction, but this resulted in 24% (n=9) of responders changing their preference to an emergency department transfer. As for vascular impairment, 45% (n=17) continued to opt for medical room reduction, but this led to 21% (n=8) of responders changing their preference to an on-pitch reduction. These responses contrast with advice from Shah et al. and Norte et al., whereby all dislocations with neurovascular impairment should be immediately transferred to an emergency department [4,18]. Without qualitative data, these patterns are somewhat difficult to interpret, but this variability may be attributed to ambiguity within current guidelines or the spectrum of clinician experience levels, and highlights that clarification and consensus are needed to optimise management.

The reduction technique was an area of discrepancy amongst responders. The Spaso method was the most popular first reduction technique at 42% (n=16), which is consistent with PHICIS course recommendations [16] and is included within the suggested methods of Shah et al. [4] and Norte et al. [18]. But numerous other techniques were reported, highlighting the plethora of options and absence of strong consensus. Clinicians opted to have a mean of 1.6 and 1.1 attempts at reduction for the initial and second methods, respectively. However, with 46% (n=17) having more than one attempt at a first reduction method, and 87% (n=33) having more than one total reduction attempts, this appears inconsistent with advice from PHICIS course recommendations and Shah et al. which both recommend one reduction attempt of one method before transfer to an ED [4,10], and Norte et al. which suggests two attempts of one reduction method [18]. Although clinicians can work in different settings and have different experiences and preferences, guidelines and pitch-side courses should aim to give more clarification on optimal reduction technique and the number of recommended attempts in the pitch-side setting.

Pitch-side courses did appear to be influential in some questions, such as neurovascularly intact reduction location and reduction method, which likely reflects their mandatory status in professional rugby healthcare. However, as previously discussed, courses may not necessarily be evidence-based and may contain insufficient detail regarding the complexities of shoulder dislocation pitch-side management, which is highlighted by inconsistencies in other management areas.

At the time of writing, this study appears to be the first of its kind in professional rugby union and sport worldwide. Internationally, and outside of professional rugby union, it remains unknown whether there is variation in pith-side sport shoulder dislocation management due to a paucity of research and currently no American, Australian, New Zealand or other orthopaedic or sports medicine guidelines at present [4,43]. The study findings cannot be extrapolated outside of professional rugby union or the UK and Ireland but may be useful for awareness of practices and reflection amongst international and other sporting pitch-side clinicians.

Strengths and limitations

A key strength of this study was the use of a multidisciplinary expert panel for questionnaire development. The panel included professionals across physiotherapy, upper limb orthopaedics, emergency medicine, sport and exercise medicine, research, and the RFU medical director, which provided a broad, relevant range of clinical perspectives. This strengthened the content validity of the questionnaire and ensured it was informed by current practice across multiple relevant domains. Questionnaire distribution was also a notable strength. It reached 41 out of 51 professional rugby teams (80%) across the UK and Ireland via official channels - the RFU and IRFU - and was supported and endorsed by the RFU medical director, which facilitated access to a difficult-to-reach population.

However, several limitations must be acknowledged. The final response count (n=38) did not meet the calculated sample size of 142, limiting the study’s statistical power and representativeness. The overall response rate of 27% also reduces the external validity and generalisability of the findings. This is a recognised limitation of survey-based research, particularly within professional sport, where clinicians often face significant time and performance pressures [44]. Although efforts were made to maximise responses-such as formal distribution via national governing bodies, reminder emails, and anonymous participation-non-response bias remains a possibility. Likely, those with a particular interest or experience in shoulder dislocation management were more inclined to respond, potentially skewing the results. Nevertheless, the diversity of respondents across different professional roles and clinical experience suggests that the study still captured a broad and informative cross-section of current practice.

Another limitation was the absence of representation from clinicians working in Scottish and Welsh professional rugby unions. The survey was not distributed by the Scottish Rugby Union (SRU) or Welsh Rugby Union (WRU), which precludes full UK-wide generalisability. Additionally, while the survey targeted the head medical staff, the use of an online questionnaire meant that responder identity and credentials could not be independently verified. There is also the possibility that multiple respondents from the same club participated, potentially introducing response clustering and limiting the independence of responses.

The survey validation process was limited to a single round of expert feedback. Ideally, multiple iterative rounds would have allowed for refinement and stronger consensus. Furthermore, while the survey captured descriptive data effectively, it did not collect qualitative responses. As such, the reasoning behind clinical decisions, as well as contextual factors influencing practice, could not be fully explored. Finally, this study used a non-randomised, convenience sampling approach due to the practical challenges of accessing professional rugby clinicians. While this allowed for efficient data collection, it may introduce selection bias and limit generalisability to all medical personnel within the sport.

Future directions

This study was designed as an exploratory investigation into pitch-side management practices for anterior shoulder dislocation in professional rugby union - an area with minimal published literature, clinical guidance, or standardised protocols. A descriptive methodology was intentionally used to provide an initial overview of clinician behaviour and to identify key areas of both consistency and variation across professional settings. By avoiding statistical hypothesis testing - particularly given the small sample size - the study aimed to reduce confirmation bias and instead lay a foundation for future, more robust research. These findings may serve as a springboard for hypothesis-driven studies aimed at developing evidence-based clinical pathways and improving governance in pitch-side care, both within rugby and other contact sports.

Further research should explore the impact of specific training programs on clinical decision-making. Comparing outcomes between clinicians trained in different educational programmes - such as PHICIS, SCRUMCAPS, or other sport-specific initiatives - could provide insight into which courses best prepare clinicians for effective pitch-side management. Evaluating these training pathways would support the development of more targeted, evidence-informed educational content and ultimately enhance clinician preparedness across levels of play.

Given that this study did not assess clinical outcomes, future investigations should focus on the efficacy and safety of different reduction techniques, as well as clinician preferences, confidence levels, and patient outcomes. This would allow for a more comprehensive understanding of the benefits and risks of various techniques and how they are influenced by factors such as professional background, training history, and available resources.

Lastly, broader research is needed to evaluate shoulder dislocation management across other sports and geographic regions. Comparative data between rugby and other collision or contact sports could uncover sport-specific challenges, while cross-national comparisons may help guide the creation of universally applicable or context-specific guidelines. The development of a robust, standardised set of pitch-side guidelines - grounded in evidence and reflective of real-world clinical environments - remains a clear priority for future research and policy development.

Clinical implications

This research appears to be the first study evaluating initial management practices of anterior shoulder dislocation management within professional UK and Ireland rugby union and professional sport in general. This work provides an understanding of current management, which is highly valuable for several groups, including clinicians, researchers, pitch-side course teachers, and national rugby governing bodies. Clinicians may reflect on current practice, leading to either an increased confidence in their current approach or a slight adjustment of practices if necessary. Pitch-side course teachers may use the findings to guide course discussions with more clarity and highlight current practice at the professional level. Researchers may use this study as a basis to compare practices across countries or to further study management decisions and their effect on injury burden, return to sport, and complication rate.

## Conclusions

Anterior shoulder dislocation is a common injury in professional rugby union, and its pitch-side management is critical to athlete care. This exploratory study - the first of its kind in this setting - identified both consistency and notable variation in current practices, particularly in reduction settings for neurovascular impairments, reduction techniques, and the number of attempts. While pitch-side courses and national guidelines influence practice, the lack of standardised, evidence-based protocols and internal club guidelines highlights a clear need for greater clarity and consensus. Although the response rate limits generalisability, the inclusion of a range of respondents captured a meaningful snapshot of current clinical behaviour. These findings offer a foundation for future research into outcomes, training effectiveness, and management strategies. To support more consistent and high-quality care, the development of robust, evidence-informed pitch-side guidelines is recommended in the field of professional rugby in the UK and Ireland.
